# Cardiac remodeling following reperfused acute myocardial infarction is linked to the concomitant evolution of vascular function as assessed by cardiovascular magnetic resonance

**DOI:** 10.1186/s12968-016-0314-6

**Published:** 2017-01-04

**Authors:** Olivier Huttin, Damien Mandry, Romain Eschalier, Lin Zhang, Emilien Micard, Freddy Odille, Marine Beaumont, Renaud Fay, Jacques Felblinger, Edoardo Camenzind, Faïez Zannad, Nicolas Girerd, Pierre Y. Marie

**Affiliations:** 1 CHRU-Nancy, Department of Cardiology, Nancy, F-54000 France; 2 INSERM, UMR-1116, Nancy, F-54000 France; 3 INSERM, UMR-947, Nancy, F-54000 France; 4 CHRU-Nancy, Department of Radiology, Nancy, F-54000 France; 5 Faculty of Medicine, Université de Lorraine, Nancy, F-54000 France; 6 CHU-Clermont-Ferrand, Department of Cardiology, Clermont-Ferrand, F-63000 France; 7 Université d’Auvergne, UMR6284, Clermont-Ferrand, F-63000 France; 8 INSERM CIC 1433, Nancy, F-54000 France; 9 CHRU-Nancy, Hôpitaux de BRABOIS, Service de Médecine Nucléaire, Allée du Morvan, 54500 Vandœuvre, France

**Keywords:** Hemodynamics, Myocardial infarction, Cardiac remodeling, Peripheral vascular resistance, Cardiovascular magnetic resonance, CMR

## Abstract

**Background:**

Left ventricular (LV) remodeling following acute myocardial infarction (MI) is difficult to predict at an individual level although a possible interfering role of vascular function has yet to be considered to date. This study aimed to determine the extent to which this LV remodeling is influenced by the concomitant evolution of vascular function and LV loading conditions, as assessed by phase-contrast Cardiovascular Magnetic Resonance (CMR) of the ascending aorta.

**Methods:**

CMR was performed in 121 patients, 2–4 days after reperfusion of a first ST-segment elevation myocardial infarction and 6 months thereafter. LV remodeling was: (i) assessed by the 6-month increase in end-diastolic volume (EDV) and/or ejection fraction (EF) and (ii) correlated with the indexed aortic stroke volume (mL.m^−2^), determined by a CMR phase-contrast sequence, along with derived functional vascular parameters (total peripheral vascular resistance (TPVR), total arterial compliance index, effective arterial elastance).

**Results:**

At 6 months, most patients were under angiotensin enzyme converting inhibitors (86%) and beta-blockers (84%) and, on average, all functional vascular parameters were improved whereas blood pressure levels were not. An increase in EDV only (EDV+/EF-) was documented in 17% of patients at 6 months, in EF only (EDV-/EF+) in 31%, in both EDV and EF (EDV+/EF+) in 12% and neither EDV nor EF (EDV-/EF-) in 40%. The increase in EF was mainly and independently linked to a concomitant decline in TPVR (6-month change in mmHg.min.m^2^.L^−1^, EDV-/EF-: +1 ± 8, EDV+/EF-: +3 ± 9, EDV-/EF+: -7 ± 6, EDV+/EF+: -15 ± 20, *p* < 0.001) while the absence of any EF improvement was associated with high persisting rates of abnormally high TPVR at 6 months (EDV-/EF-: 31%, EDV+/EF-: 38%, EDV-/EF+: 5%, EDV+/EF+: 13%, *p* = 0.007). By contrast, the 6–month increase in EDV was mainly dependent on cardiac as opposed to vascular parameters and particularly on the presence of microvascular obstruction at baseline (EDV-/EF-: 37%, EDV+/EF-: 76%, EDV-/EF+: 38%, EDV+/EF+: 73%, *p* = 0.003).

**Conclusion:**

LV remodeling following reperfused MI is strongly influenced by the variable decrease in systemic vascular resistance under standard care vasodilating medication. The CMR monitoring of vascular resistance may help to tailor these medications for improving vascular resistance and consequently, LV ejection fraction.

**Trial registration:**

NCT01109225 on ClinicalTrials.gov site (April, 2010).

## Background

Left ventricular (LV) remodeling following acute myocardial infarction (MI) is a complex process possibly leading in the first months to an improvement in ejection fraction (reverse remodeling [1–3]) and to an increased end-diastolic volume (adverse remodeling [1, 4, 5]). This remodeling is difficult to predict at an individual level although a possible interfering role of vascular function has never been considered to date.

Cardiovascular Magnetic resonance (CMR) has gained a pivotal role in assessing LV function and for characterizing MI areas in this setting [4, 6]. Aortic stroke volume (SV), along with derived functional vascular parameters, can also be assessed with phase-contrast CMR, independently of LV volume values and with an accuracy level that is not reached by Doppler techniques [7].

The combined analysis of brachial blood pressure (BP) and of the SV producing the BP wave has been recently shown to significantly improve the CMR characterization of LV remodeling in hypertensive patients [8, 9]. This improvement is notably due to the precise information obtained on LV loading conditions with the non-invasive determination of various parameters such as total peripheral vascular resistance, total arterial compliance index and effective arterial elastance [8–13]. However, this vascular monitoring has yet to be investigated in the context of LV remodeling following reperfused acute MI and where patients are commonly referred to hypotensive medical regimens (angiotensin converting enzyme (ACE) inhibitors, beta-blockers). These medications are indeed likely to influence blood pressure and vascular function and thereby, LV wall stress, a main stimulus for adverse LV remodeling [1].

In light of the above, the present study was aimed at determining the extent to which vascular function and LV loading conditions, assessed through an aortic phase-contrast CMR sequence, and their changes over time influences LV remodeling following reperfused MI.

## Methods

### Study population

The study population was extracted from the single-center “REMI” (relation between aldosterone and cardiac REmodeling after Myocardial Infarction) cohort in which serial CMR and biomarker measurements were performed after reperfused acute MI [14, 15]. The study protocol, in compliance with the Declaration of Helsinki, was approved by the local Ethics Committee (CPP agreement n° 2009-A00537-50) and was registered on the ClinicalTrials.gov site (NCT01109225). All subjects gave signed informed consent to participate.

Patients successfully treated by primary percutaneous transluminal coronary angioplasty for a first ST-segment elevation myocardial infarction (STEMI) were prospectively included. In order to be retained, acute STEMI had to be associated with a significant rise in cardiac enzymes and with an initial occlusion or sub-occlusion of the MI-related artery at angiography (Thrombolysis In Myocardial Infarction flow grade 0 to 1). Main exclusion criteria were: (i) previous history of MI, (ii) any other significant cardiac disease (including > grade-2 mitral regurgitation), (ii) any contraindication to CMR, (iii) absence of sinus cardiac rhythm, (iv) a multivessel disease at coronary angiography and (v) a >12 h delay-time between the onset of chest pain and reperfusion. The patients were referred to a medical check-up involving CMR at 2 to 4 days after acute MI reperfusion and 6 months (±15 days) later.

### CMR

CMR was performed on a single 3 T magnet (Signal HDxt, GE Healthcare, Milwaukee, Wisconsin) equipped with a dedicated cardiac coil. Systolic, diastolic and mean brachial blood pressures (BP) were measured with an automated sphygmomanometer (Maglife C, Schiller Medical, Wissembourg, France). Three measurements were obtained during the CMR examination and mean values were stored for analyses herein.

A steady-state free precession pulse sequence was used to assess LV function in contiguous short axis planes. Each plane was recorded as previously detailed [8, 9, 16], and LV end-diastolic volume (EDV), end-systolic volume and ejection fraction (EF) were obtained by an expert cardiologist, using dedicated software (MASS research v2013-exp™, Medis, Leiden University Medical Center, The Netherlands). Main acquisition parameters were as follows: 8 mm slice-thickness, 3.5–3.9 ms repetition time, 14 to 16 K-space lines per segment, 1.5 ASSET factor, 30 phases per cardiac cycle with view sharing, field-of-view (FOV) ranging from 32 to 38 cm with a phase FOV of 0.9, and a 224x224 matrix interpolated to 256x256.

Flow was determined in the ascending aorta using a velocity-encoded phase-contrast gradient-echo sequence and with the “CV flow” software (Leiden University Medical Center, Medis, The Netherlands) [8, 9]. Velocities were corrected by a ROI-based method only in instances of evident offset error [7]. Acquisition parameters were as follows: 8 mm slice-thickness, 10° flip angle, 3–4 ms echo time, 6–7 ms repetition time, 31 kHz bandwidth, 6 K-space lines per segment, 32 phases per cardiac cycle with view sharing, unidirectional velocity encoding with a maximal velocity set to 150 cm.sec^−1^, FOV between 30 to 38 cm with a phase FOV of 0.85, and a 256x128 matrix interpolated to 256x256. Aortic stroke volume (SV) was indexed to body surface area and used to calculate the following [8, 9]: cardiac index (SV x heart rate), total arterial compliance index (TAC: SV/pulse pressure), effective arterial elastance (Ea: mean BP/SV), stroke work (mean BP x SV) and total peripheral vascular resistance (TPVR: mean BP/cardiac index). TPVR values above 40 mmHg.min.m^2^.L^−1^ were considered as abnormally high, a threshold corresponding to the upper limit of the 95% confidence interval in an already-described normal population of 100 subjects with comparable age range and CMR protocol as in the population of subjects in the current study [9].

The MI area was analyzed on 8 to 10 short axis slices covering the LV volume and two vertical and horizontal long-axis slices, which were recorded with a T1-weighted segmented phase-sensitive inversion-recovery (PSIR) sequence 10 to 15 min after the injection of a gadolinium-labeled tracer (0.1 mmol.kg^−1^ of body weight of Dotarem®, GUERBET, France) with the following parameters: 4.7/2.1 msec for TR/TE, a typical 250–350 msec inversion time adjusted to null the signal of the non-infarcted myocardium, 28–35 cm field-of-view, 192x128 matrix interpolated to 256x256, 8 mm slice-thickness and 20° flip angle.

The MI volume was considered as that showing a late gadolinium enhancement (LGE) by visual analysis and expressed in % of the total LV volume by using a 17-segment LV division and while taking into account the number of quartiles involved in each segment [16, 17]. The size of the transmural MI was determined as the % of LV segments showing a LGE ≥75% of myocardial thickness, while its involvement in microvascular obstruction was determined as that showing a central hypo enhancement within the bright signal [4].

Intra-observer reproducibility of the determinations of LV-volumes and EF was assessed in 30 consecutive CMR exams from the present cohort which were analyzed twice in an interval greater than 1 month apart. Absolute values of the relative variations between the first and second measurements were on average 6.59 ± 5.33 mL for EDV and 2.95 ± 2.65% for EF.

### Statistical analysis

LV remodeling was assessed by the increases in EDV and EF between baseline and 6 months, and the corresponding thresholds were determined as the upper limits of the 95% confidence interval of the reproducibility analysis described above, i.e. >17.3 mL for EDV and >8.3% for EF.

Continuous variables are expressed as means ± standard deviation [SD] and discrete variables as percentages. Paired comparisons between baseline and 6 months were performed with Wilcoxon tests for continuous variables and McNemar tests for percentages. Unpaired comparisons of continuous variables were performed with Mann-Whitney tests when comparing two groups and with Kruskal-Wallis tests for more than 2 groups. Unpaired intergroup comparisons of discrete variables were performed with chi-square tests or Fisher's exact tests where appropriate. Significant univariate predictors of an increase in EDV or in EF at 6 months were investigated among the baseline variables listed in Tables 1 and 2 as well as among the 6-month changes in vascular parameters (BP, TPVR, TAC and Ea). In all above tests, a 2-sided *p*-value < 0.05 was considered statistically significant.

**Table 1:** Main general characteristics of the study population at baseline (*n* = 121)

Age (years)	56 ± 10
Female gender	18 (15%)
Hypertension	39 (32%)
Diabetes Mellitus	10 (8%)
Hypercholesterolemia	47 (39%)
Obesity	9 (7%)
Peak Troponin-Ic (ng/ml)	922 ± 2014
Primary angioplasty	
Use of glycoprotein IIb/IIIa inhibitor	77 (64%)
Delay from pain onset (hours)	4.3 ± 2.3
MI-related coronary artery	
- left anterior descending artery	65 (54%)
- right coronary artery	45 (37%)
- left circumflex artery	11 (9%)
Stent implantation	117 (97%)

**Table 2:** Main parameters recorded at baseline and at 6 months with *p* values for significant paired comparisons

	Baseline	6 months	*p*-value
Main medications			
- Beta-blockers	100 (83%)	101 (84%)	
- ACE inhibitors	103 (85%)	104 (86%)	
- Statins	110 (91%)	105 (87%)	
- Antiplatelet therapy	120 (99%)	115 (95%)	
Body mass index (kg.m^−2^)	25 ± 4	25 ± 4	
Heart rate (bpm)	66 ± 11	57 ± 8	< 0.001
BP parameters			
Systolic BP (mmHg)	129 ± 21	130 ± 20	
Diastolic BP (mmHg)	75 ± 14	72 ± 11	
Mean BP (mmHg)	93 ± 15	92 ± 13	
Cardiac parameters			
LV end-diastolic volume (mL.m^−2^)	92 ± 15	96 ± 18	0.006
LV ejection fraction (%)	42 ± 8	49 ± 9	< 0.001
Total MI area (% of LV)	22 ± 12	16 ± 11	< 0.001
Transmural MI - presence (%)	94 (78%)	55 (46%)	< 0.001
- area (% of LV)	16 ± 13	8 ± 11	< 0.001
MVO - presence (%)	59 (49%)	0 (0%)	< 0.001
- area (% of LV)	8 ± 11	0 ± 0	< 0.001
SV-derived parameters			
SV (mL.m^−2^)	38 ± 8	47 ± 9	< 0.001
Cardiac index (L.min^−1^.m^−2^)	2.4 ± 0.4	2.6 ± 0.5	< 0.001
TPVR (mmHg.min.m^2^.L^−1^)	39 ± 11	36 ± 9	0.001
TAC (mL.m^−2^.mmHg^−1^)	0.74 ± 0.21	0.83 ± 0.22	< 0.001
Ea (mmHg.m^2^.mL^−1^)	2.6 ± 0.9	2.1 ± 0.6	< 0.001
Stroke Work (L.mmHg.m^−2^)	3.5 ± 0.9	4.3 ± 1.0	< 0.001

*ACE* angiotensin converting enzyme, *BP* blood pressure, *Ea* effective arterial elastance, LV left ventricular, *MI* myocardial infarction, *MVO* microvascular obstruction, *SV* stroke volume, *TAC* total arterial compliance, *TPVR* total peripheral vascular resistance

Multivariable stepwise logistic regressions were also applied for predicting an increase in EDV or in EF at 6 months, with backward-forward selection among significant univariate predictors and with *p*-values of 0.05 and 0.10 for entering and removing the variables, respectively.

## Results

### Patient characteristics at baseline

A total of 141 patients were included, among whom 20 had no CMR at 6 months (three with contraindications and 17 consent withdrawals), yielding a total of 121 patients for the present analysis. Baseline characteristics are detailed in Tables 1 and 2. Mean age was 56 ± 10 years, 17% were women, the MI-related vessel was the left anterior descending artery in 54% of cases and primary angioplasty was performed on average 4.3 ± 2.3 h after the onset of chest pain.

At baseline CMR, mean LV ejection fraction (EF) was 42 ± 8%, 98% of patients exhibited an MI area with LGE while 49% had microvascular obstruction.

### Evolution at 6 months

Paired comparisons between baseline and 6 months are detailed in Table 2. Most patients were still under beta-blocker treatment (84%) at 6 months, as well as under angiotensin enzyme converting (ACE) inhibitor (86%) although only 52% of the overall population had an equivalent dose corresponding to that targeted in post-MI trials [18]. Only six patients received mineralocorticoid receptor antagonist treatment and only two had vasodilators other than ACE inhibitors.

On average, marked improvements were observed from baseline to 6 months for all SV-derived parameters, including TPVR, TAC and Ea, whereas BP levels remained unchanged. An abnormally high TPVR (>40 mmHg.min.m^2^.L^−1^) was documented in as many as 56 patients at baseline (46%) and was still present in 27 (22%) at 6 months (*p* < 0.001). These 27 patients with a persistent high TPVR had a mean systolic blood pressure of 144 ± 19 mmHg during the CMR exam, with slightly over half (52%) under the level of 150 mmHg.

Most LV function parameters were also increased, on average, namely EF, EDV, stroke volume, stroke work and cardiac index (Table 2). Significant increases in EDV only were documented in 21 patients (17%), in EF only in 37 (31%), in both EDV and EF in 15 (12%) and in neither EDV nor EF in 48 (40%).

### Predictions of the increases in EDV and in EF

As detailed in Table 3, among baseline cardiac variables, those reflecting MI severity (areas with LGE, microvascular obstruction), were strongly correlated with a 6-month increase in EDV, whereas patients with an increase in EF were rather characterized by a lower baseline EF.

**Table 3:** Mean (± SD) and *p* values for the significant univariate correlates of the two-group comparisons between patients with and those without 6-month increases in LV end-diastolic volume (EDV, left panel) or in ejection fraction (EF, right panel)

	Increase in EDV			Increase in EF		
	Presence (*n* = 36)	Absence (*n* = 85)	*p*-value	Presence (*n* = 52)	Absence (*n* = 69)	*p*-value
Baseline parameters						
Delay from pain onset (hours)	4.9 ± 2.9	4.0 ± 1.9	NS	4.4 ± 2.3	4.1 ± 2.3	NS
MI-related coronary artery						
- left anterior descending artery	24 (67%)	41 (48%)	NS	29 (56%)	36 (52%)	NS
- right coronary artery	10 (28%)	35 (41%)	NS	20 (38%)	25 (36%)	NS
- left circumflex artery	2 (6%)	9 (11%)	NS	3 (6%)	8 (12%)	NS
Heart rate (bpm)	69 ± 12	64 ± 10	0.037	67 ± 12	65 ± 9	NS
LV ejection fraction (%)	40 ± 8	43 ± 8	0.041	39 ± 8	44 ± 7	<0.001
Total MI area (% of LV)	25 ± 12	20 ± 11	0.022	22 ± 12	22 ± 11	NS
Transmural MI area (% of LV)	21 ± 13	14 ± 12	0.007	16 ± 12	16 ± 13	NS
MVO - presence (%)	27 (75%)	32 (38%)	<0.001	25 (48%)	34 (49%)	NS
- area (% of LV)	13 ± 12	6 ± 10	0.001	8 ± 10	8 ± 11	NS
SV (mL.m^−2^)	35 ± 8	39 ± 7	0.011	36 ± 8	39 ± 8	0.023
TPVR (mmHg.min.m^2^.L^−1^)	41 ± 16	38 ± 8	NS	42 ± 13	37 ± 7	0.014
TAC (mL.m^−2^.mmHg^−1^)	0.72 ± 0.22	0.75 ± 0.21	NS	0.69 ± 0.20	0.78 ± 0.211	0.021
Ea (mmHg.m^2^.mL^−1^)	2.9 ± 1.2	2.5 ± 0.6	0.044	2.8 ± 1.1	2.4 ± 0.6	0.009
Stroke work (L.mmHg.m^−2^)	3.1 ± 0.7	3.6 ± 0.9	0.006	3.4 ± 0.9	3.5 ± 0.8	NS
Changes in vascular parameters from baseline to 6 months						
Diastolic BP (mmHg)	−2 ± 17	−3 ± 14	NS	−7 ± 14	0 ± 14	0.011
Mean BP (mmHg)	1 ± 21	−2 ± 15	NS	−5 ± 17	2 ± 16	0.033
TPVR (mmHg.min.m^2^.L^−1^)	−5 ± 17	−3 ± 8	NS	−10 ± 12	1 ± 9	<0.0001
TAC (mL.m^−2^.mmHg^−1^)	0.1 ± 0.3	0.1 ± 0.2	NS	0.2 ± 0.2	0.0 ± 0.2	0.001
Ea (mmHg.m^2^.mL^−1^)	−0.8 ± 1.2	−0.4 ± 0.6	0.032	−0.9 ± 0.9	−0.2 ± 0.6	<0.0001

*NS* non-significant; see Table 2 for the other abbreviations

The 6-month increases in EDV and EF were additionally predicted by a lower baseline SV (Table 3), and among all SV-derived parameters, a lower baseline stroke work was associated with an increase in EDV, whereas worse functional vascular parameters at baseline (i.e. higher Ea and TPVR and lower TAC) were associated with an increase in EF. Finally, a 6-month improvement in these vascular parameters was a main characteristic of patients with an increase in EF (Table 3) with an especially notable decrease in TPVR between baseline and 6 months (in mmHg.min.m^2^.L^−1^: −10 ± 12 for patients with EF-increase vs. +1 ± 9 for the others, *p* < 0.001).

The strongest multivariate correlates for the 6-month increase in EF were the concomitant change in TPVR (*p* < 0.001) and baseline EF (*p* = 0.028) (global *R* ^2^ = 0.40), while the strongest correlates for the 6-month increase in EDV were baseline stroke work (*p* = 0.019) and presence of microvascular obstruction at baseline (*p* = 0.001) (global *R* ^2^ = 0.21).

**Fig. 1:**
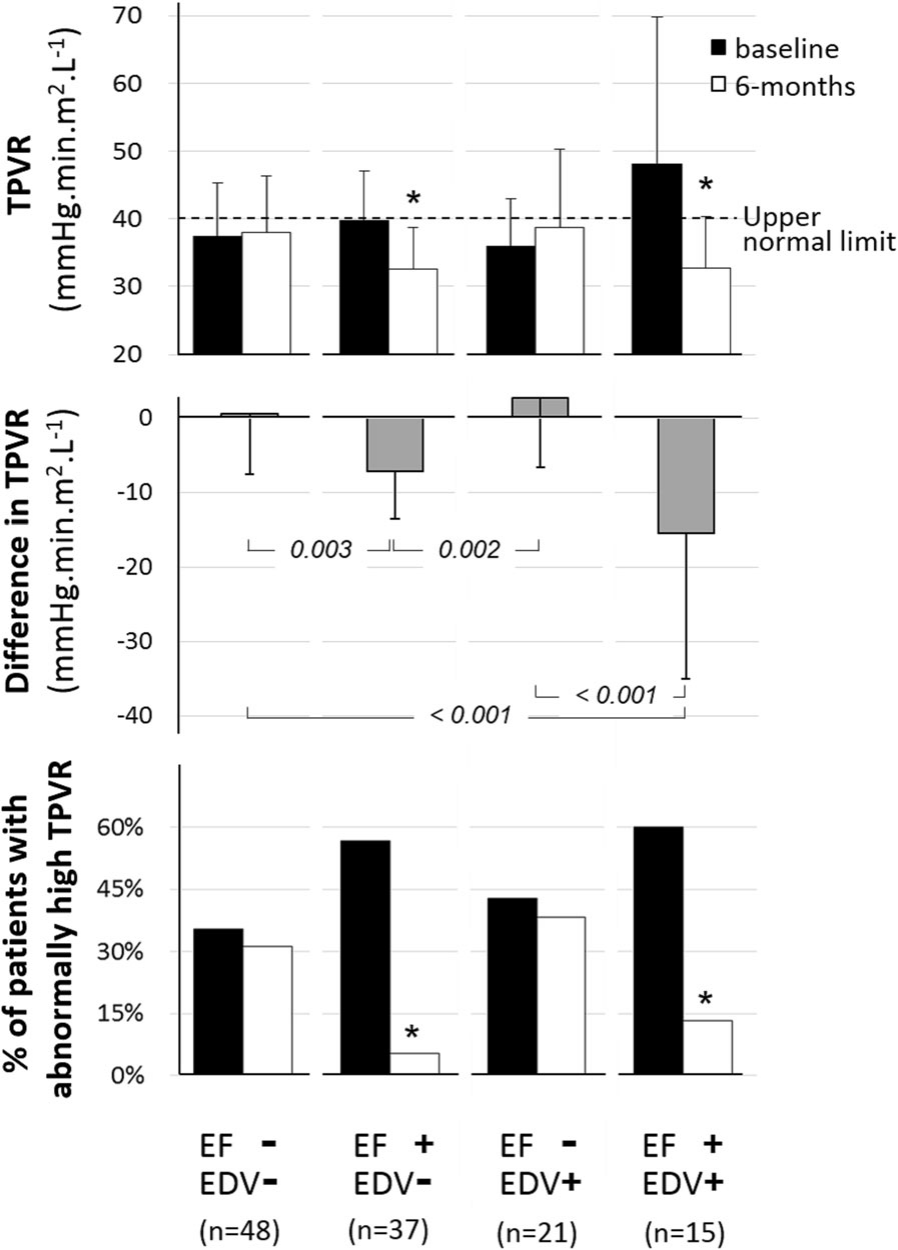
Mean values (±SD) for baseline (*black* columns) and 6-month (*white* columns) values of total peripheral vascular resistance (TPVR, *upper panel*), for the mean difference in TPVR between baseline and 6 months (*grey* columns, median panel) and for the percentages of patients with abnormally high TPVR (>40 mmHg.min.m^2^.L^−1^, *lower panel*) in patients categorized in 4 groups: (i) those without any significant increase in EF or EDV at 6 months (EF-/EDV-), (ii) those with an increase in EF only (EF+/EDV-) or (iii) EDV only (adverse remodeling, EF-/EDV+), and (iv) those with increase in both EF and EDV (EF+/EDV+). *: *p* < 0.05 for paired comparisons between baseline and 6 months

**Fig. 2:**
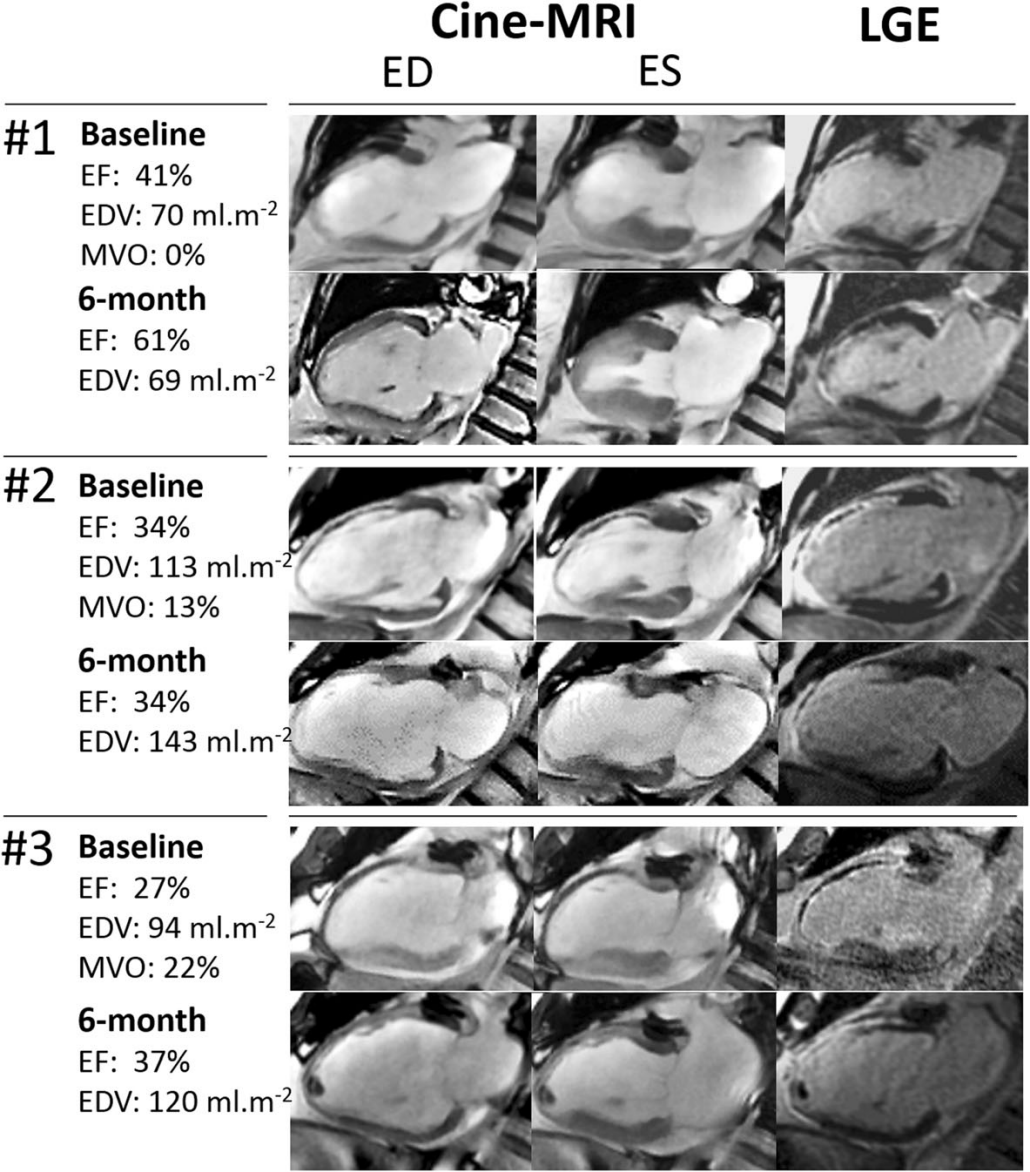
Mid-ventricular vertical long-axis slices at baseline and at 6 months obtained from late gadolinium enhancement (LGE) images (*right panel*) and cine-MRI at end-diastole (ED, *left panel*) and end-systole (ES, *middle panel*), with LV parameters (EF for ejection fraction; EDV for end diastolic volume and MVO for % of LV area with microvascular obstruction), in 3 representative cases of anterior infarction and 6-month increase(s): #1) in EF only and a 6-month decrease in TPVR (-8 mmHg.min.m^2^.L-1), #2) in EDV only and no clear change in TPVR (-1 mmHg.min.m^2^.L-1) and #3) in both EDV and EF and a 6-month decrease in TPVR (-11 mmHg.min.m^2^.L-1)

Figure 1 provides an illustration of the comparative evolution of TPVR from baseline to 6 months between the four groups of patients with increases in EDV only (EDV+/EF-), EF only (EDV-/EF+), both EDV and EF (EDV+/EF+) and neither EDV nor EF (EDV-/EF). Representative cases are also shown in Fig. 2. The two main observations are that i) the increase in EF was strongly linked to a concomitant decline in TPVR (6-month change in mmHg.min.m^2^.L^−1^, EDV-/EF-: +1 ± 8, EDV+/EF-: +3 ± 9, EDV-/EF+: -7 ± 6, EDV+/EF+: -15 ± 20, *p* < 0.001) and ii) the absence of EF improvement was associated with high persistent rates of abnormally high TPVR at 6 months (EDV-/EF-: 31%, EDV+/EF-: 38%, EDV-/EF+: 5%, EDV+/EF+: 13%, *p* = 0.007).

Lastly, of particular note, the 6–month increase in EDV was mainly dependent on cardiac as opposed to vascular parameters and particularly on the presence of microvascular obstruction at baseline, as strengthened by the following rates in the four groups: EDV-/EF-: 37%, EDV+/EF-: 76%, EDV-/EF+: 38%, EDV+/EF+: 73% (*p* = 0.003).

## Discussion

**Fig. 3:**
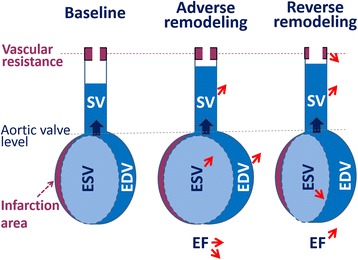
Schematic representation of the influence of the decrease in systemic vascular resistance in the mechanisms leading to reverse remodeling (where stroke volume (SV) may be improved owing to an increase in ejection fraction (EF) with a decrease in end-systolic volume (ESV)) but not to adverse remodeling (where SV improvement is attributable to an increase in end-diastolic volume (EDV) in link with MI severity and especially of microvascular obstruction). Note that adverse and reverse remodeling correspond to different mechanisms and may thus coexist in the same patients

LV remodeling following acute MI was previously shown to be difficult to predict at an individual level, although a possible interfering role of the concomitant evolution of vascular function has never been considered to date. This remodeling may be regarded as an adaptive mechanism for maintaining cardiac stroke volume after the loss of contractile tissue [1]. Indeed, as schematically represented in Fig. 3, stroke volume can be restored by an increase in LV ejection fraction but also in end-diastolic volume [1]. However, this increase in end-diastolic volume is known to result in deleterious loading conditions, promoting further enlargement and dysfunction, and thereby resulting in an adverse vicious cycle [1].

In the present study, SV-derived parameters were selected as independent predictors of LV remodeling. One such parameter is the indexed stroke work which together with the presence of microvascular obstruction provided the strongest multivariate correlation with the 6-month increase in end-diastolic volume. Indexed stroke work, calculated by the product of mean BP and indexed SV, provides an indication of the aortic flow that can potentially be generated by the LV at sufficiently high pressure and has been indubitably recognized as a strong prognostic indicator in patients with severe MI and/or heart failure [19, 20].

Microvascular obstruction, also known as “no reflow”, is currently documented at short term after an acute MI, attaining its maximal size after 48 h [4]. In accordance with previous comparable studies, the present findings show that microvascular obstruction was a strong predictor of a subsequent adverse remodeling (as defined by an increase in LV volume [4, 21–23]), but not of the evolution of LV ejection fraction [3]. The latter was rather best predicted by systemic vascular resistance, another SV-derived parameter reflecting LV afterload. More precisely, the 6-month change in TPVR was selected as the strongest independent correlate of the concomitant enhancement in LV ejection fraction, together with the baseline level of ejection fraction, with these two variables accounting for up to 40% (corresponding to the *R* ^2^ value) of the significant increases in ejection fraction at 6 months.

By contrast, BP and its changes over time were not found to be accurate indices of the evolution of LV afterload in the months following acute MI. This point is best illustrated by the observation that BP was, on average, unchanged between baseline and 6 months, whereas all SV-derived parameters of arterial afterload were markedly enhanced during this same period. These parameters included TPVR and TAC, which respectively reflect the functions of distal and proximal arterial beds, as well as Ea, which could be considered as representing a more global index of vascular function [8–10].

Vascular function was likely markedly influenced in the present instance by the medical regimen prescribed after MI, especially by ACE inhibitors, which were prescribed in approximately 85% of our patients throughout the 6 months of follow-up. Other arterial vasodilators were prescribed in only two patients and no patients were on beta-blockers with vasodilating properties.

ACE inhibitors are known to favorably alter ischemic LV remodeling with hemodynamic effects, including the lowering of both TPVR and volume expansion, leading to a reduction in LV wall stress, a main stimulus for adverse remodeling [1, 24–26]. However, only half of our patients received, at 6 months, the dose prescribed for ACE inhibitors targeted in post-MI trials, in accordance with results from previous observational studies [27].

SV values were also assessed with the SSFP sequence and by subtracting end-systolic to end-diastolic LV volumes. While these SSFP-SV values were in line with those provided by the aortic phase-contrast sequence, they nonetheless differed slightly due to methodological considerations (results not shown). Moreover, these SSFP-SV values were not used in our analyses in order to avoid their interdependence with the remodeling-related changes in LV volumes which were assessed with the same LV contours. Indeed, with the use of SSFP-SV values, any change and/or error in the determination of LV contours would symmetrically affect both SV-derived and LV-remodeling variables, leading to an exaggeration of the link between these two groups of variables.

The present study furthermore shows that the decrease in TPVR represents a key element for improving LV ejection fraction, as schematically illustrated in Fig. 3, whereas LV ejection fraction remains a major prognostic indicator after MI [28]. Therefore, it may firstly be hypothesized that a decrease in TPVR might lead to enhance not only LV ejection fraction but also the prognosis of certain categories of patients. Secondly, it is likely that such an improvement in TPVR could be targeted at least in patients for whom TPVR was still abnormally high at 6 months. These patients represented as many as 30% of those who had no increase in EF and 38% of those with definite adverse remodeling (i.e. those with a 6-month increase in EDV but not in EF). Thirdly, it must be kept in mind that while the presence of high TPVR constitutes a hallmark of vascular disease, commonly leading to hypertension, a clear rise in BP is frequently lacking in instances of cardiac dysfunction [29]. Case in point, more than half of our patients with high TPVR still had normal or sub-normal systolic BP (< 150 mmHg) during the 6-month CMR exam.

Finally, it may be hypothesized that these high-TPVR patients could benefit from a change in vasodilating drug regimen with higher doses of ACE inhibitors and/or other arterial vasodilator(s) even when BP is not clearly elevated. Larger scale studies are obviously needed to determine the extent to which such a personalized therapeutic strategy, based on the non-invasive CMR monitoring of TPVR, could better orient LV remodeling toward its reverse form, as illustrated in Fig. 3. Nevertheless, this study shows that functional vascular disease is frequent in the background of acute MI, thereby limiting any further improvement in LV ejection fraction, and that this disease should be considered in a more global strategy of cardiovascular treatment and monitoring.

A certain degree of imprecision in SV measurement, related to phase offset errors, remains the main limitation for the use of phase contrast sequences, even when these errors are tentatively corrected by ROI-based methods [7]. Nevertheless, SV has been shown to be successfully assessed with MRI phase-contrast sequences with a lower than 10% error, an accuracy level unattainable by Doppler techniques [7]. Another limitation is the absence of T2*- or T2-weighted imaging for quantifying intramyocardial edema or intramyocardial hemorrhage, whereas this could have led to further improvement in the prediction of LV remodeling [4].

## Conclusions

The present study shows that LV remodeling following reperfused MI is linked to the concomitant evolution of loading conditions and particularly to the variable decrease in vascular resistance under standard care cardiovascular medication. It is likely that the non-invasive CMR monitoring of vascular resistance may help to tailor these medications in order to improve vascular resistance and consequently, LV ejection fraction.

## Abbreviations

ACE: Angiotensin converting enzyme
BP: Blood pressure
CMR: Cardiovascular magnetic resonance
Ea: Effective arterial elastance (mean BP / SV)
EDV: End-diastolic volume
EF: Ejection fraction
LGE: Late gadolinium enhancement
LV: Left ventricle
MI: Myocardial infarction
MVO: Microvascular obstruction
SV: Stroke volume
TAC: Total arterial compliance index (SV / pulse pressure)
TPVR: Total peripheral vascular resistance (mean BP / cardiac index)
